# Case Report: Long-term complications of subcutaneous ureteral bypass migration in an adult female Papillon

**DOI:** 10.3389/fvets.2025.1543299

**Published:** 2025-03-12

**Authors:** Boram Lee, Jeonghyun Seo, Soon-Wuk Jeong

**Affiliations:** ^1^Department of Veterinary Surgery, College of Veterinary Medicine, Konkuk University, Seoul, Republic of Korea; ^2^Department of Veterinary Medicine, College of Veterinary Medicine, Jeju National University, Jeju, Republic of Korea; ^3^Time Animal Medical Center, Daejeon, Republic of Korea

**Keywords:** ureteral obstruction, ureterolithiasis, hydronephrosis, foreign body reaction, Dacron cuff

## Abstract

A 6-year-old spayed female 4.4-kg Papillon with only the left kidney presented with vomiting. Imaging unveiled ureterolithiasis and hydronephrosis, while serum chemistry displayed elevated creatinine, blood urea nitrogen, and C-reactive protein. Urinalysis revealed bacteria and bacterial phagocytes. After subcutaneous ureteral bypass (SUB) placement, kidney panels were normalized. The nephrostomy and cystostomy catheters had migrated into the renal parenchyma and bladder wall on postoperative day (POD) 212 and 369, respectively. As the migration advanced, they entered the ureter and bladder on POD 369 and 796, respectively. The SUB, excluding the nephrostomy catheter, was removed on POD 930 due to migration, obstruction, and extrusion of the SUB shunting port. On POD 937, creatinine and BUN levels remained normal. By POD 1063, the ureteroliths had disappeared. This case highlights the need for vigilant monitoring of catheter migration as a potential complication following SUB placement. Early identification and timely intervention are essential for reducing morbidity and improving patient outcomes.

## 1 Introduction

Causes of ureteral obstruction include ureterolithiasis, inflammation, ureteral strictures, surgical interventions, neoplasia, and complications arising from renal transplantation (1, 2). Treatment options consist of surgical and medical management, with medical treatment demonstrating a low success rate (8–17%) (3, 4). Ureteral obstruction is considered a medical emergency, and early surgical decompression is recommended (5, 6). Traditional surgical options, such as ureterostomy, ureteral reimplantation, ureteronephrectomy, and ureteral resection with anastomosis, have been documented (4, 7). However, these methods have high complications (30–38%) and mortality rates (18–20%) (4, 8, 9). SUB is recommended for its lower mortality rate (<5%), lower complication rate (1.4–27%) and longer median survival time (762–923 days) (8, 10–12).

Long-term complications of the subcutaneous ureteral bypass (SUB), including occlusion, kinking, chronic urinary tract infection (UTI), and intermittent dysuria, have been reported (12–16). Recently, SUB migration into the gastrointestinal tract has also been reported (17–19).

This case report presents a patient in which the SUB migrated into the ureter through the renal parenchyma and the bladder. This is the first report of SUB catheter migration into both the ureter and bladder. Notably, we monitored the progression of the complication over an extended period of 1,072 days.

## 2 Case presentation

**Figure 1:**
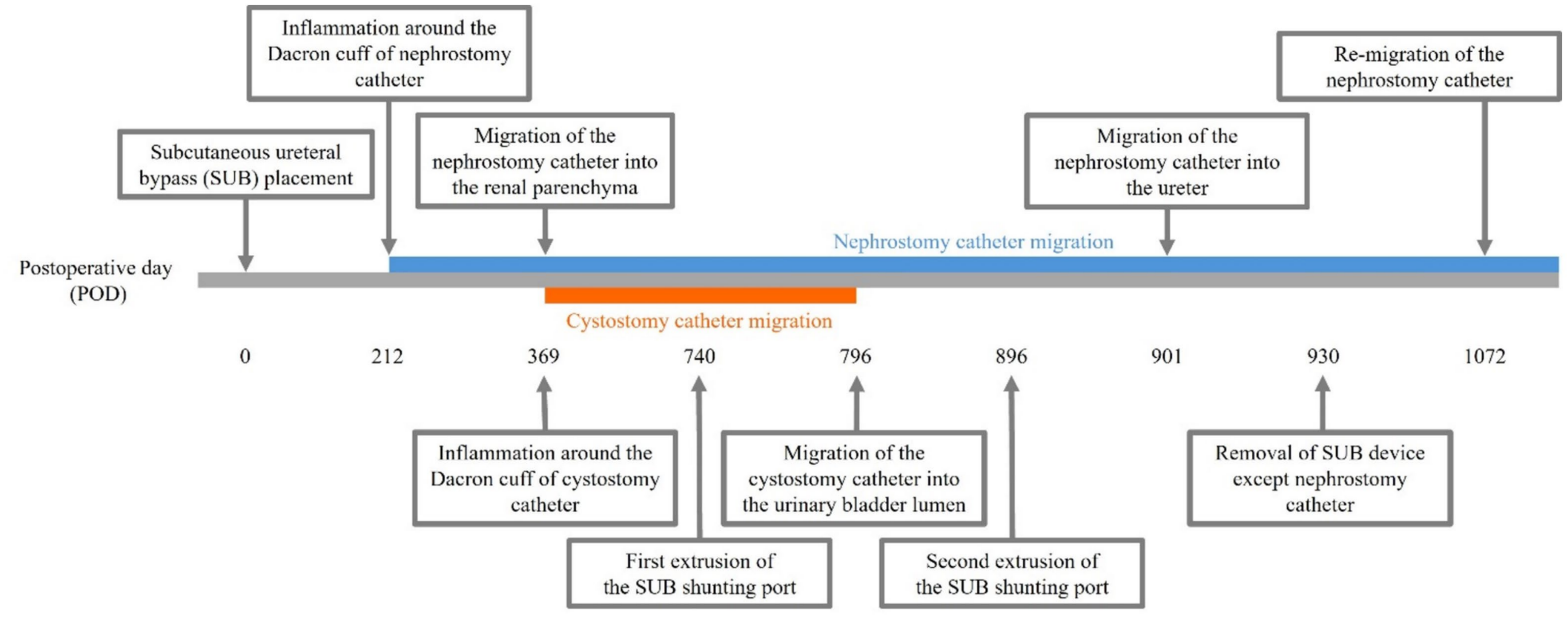
Case timeline. Inflammation around the Dacron cuff of the nephrostomy and cystostomy catheters was observed on postoperative days (POD) 212 and 369, respectively. By POD 901, the Dacron cuff of the nephrostomy catheter had gradually migrated through the renal parenchyma into the ureter, whereas by POD 796, the Dacron cuff of the cystostomy catheter had migrated into the bladder lumen. On POD 740, the subcutaneous ureteral bypass (SUB) shunting port protruded, prompting an attempt at primary wound closure. However, the SUB shunting port was re-exposed on POD 896, leading to the decision to remove the SUB device on POD 930. On POD 1072, the Dacron cuff had migrated into the renal pelvis again.

A 6-year-old spayed female 4.4-kg Papillon presented with a 2-day history of inappetence and lethargy after vomiting (Figure 1). The patient underwent a nephrectomy 5 years ago. Imaging revealed two ureteroliths (6–8 mm) in the left proximal ureter, proximal ureteral dilation, and renal pelvic dilation (8 mm) (Supplementary file 1). A physical examination revealed hyperthermia (40.9°C), and blood tests showed creatinine 2.5 mg/dL [reference interval (RI): 0.5–1.8 mg/dL], blood urea nitrogen (BUN) 38 mg/dL (RI: 7–27 mg/dL), and C-reactive protein 35.6 mg/dL (RI: 0–1 mg/dL), with no other remarkable findings. Urinalysis revealed cocci, rods, and phagocytic neutrophils. In-house antibiotic sensitivity testing revealed bacteria sensitive to enrofloxacin (Ashienro 50, Ashishi Life Science, Gujarat, India).

The patient was induced with 6 mg/kg IV propofol (Provive Inj., Pharmbio Korea, Seoul, Korea) without premedication and maintained on isoflurane (Ifran Liq., Hana Pharm, Seoul, Korea). Fluoroscope-guided placement of a SUB™ 2.0 (Norfolk Vet Products, Skokie, Illinois, United States) was conducted following a midline laparotomy. Nephrostomy and cystostomy catheters were inserted, and the Dacron cuff was secured with sterile cyanoacrylate glue and simple interrupted sutures (PDS 4–0) to the caudal pole of the kidney and the apex of the urinary bladder (Supplementary file 2). The ureteroliths remained, and the Dacron cuff was covered with perinephric fat. The catheter’s leakage and patency were verified through contrast fluoroscopy. No complications occurred during the procedure, and imaging verified successful SUB placement.

On postoperative day (POD) 1, the kidney panel (creatinine 1 mg/dL, BUN 23 mg/dL) and renal pelvic dilation improved. Following the antibiotic sensitivity tests, 2.5 mg/kg enrofloxacin (Ashienro 50, Ashishi Life Science, Gujarat, India) was prescribed orally, twice daily, for 2 months.

At 1 and 2 months postoperatively and every 1.5 months thereafter, abdominal ultrasound, serum chemistry, urinalysis, and urine cultures were conducted, followed by ultrasound-guided flushing with sterile saline and 2 mL tetra-EDTA (SUB™ Flush Kit, Norfolk Vet Products, Skokie, Illinois, United States). Prescribed targeted oral antibiotics were used, yet bacteria with comparable susceptibility profiles persisted. On POD 369, *Staphylococcus pseudintermedius* and *Escherichia coli* were identified.

**Figure 2:**
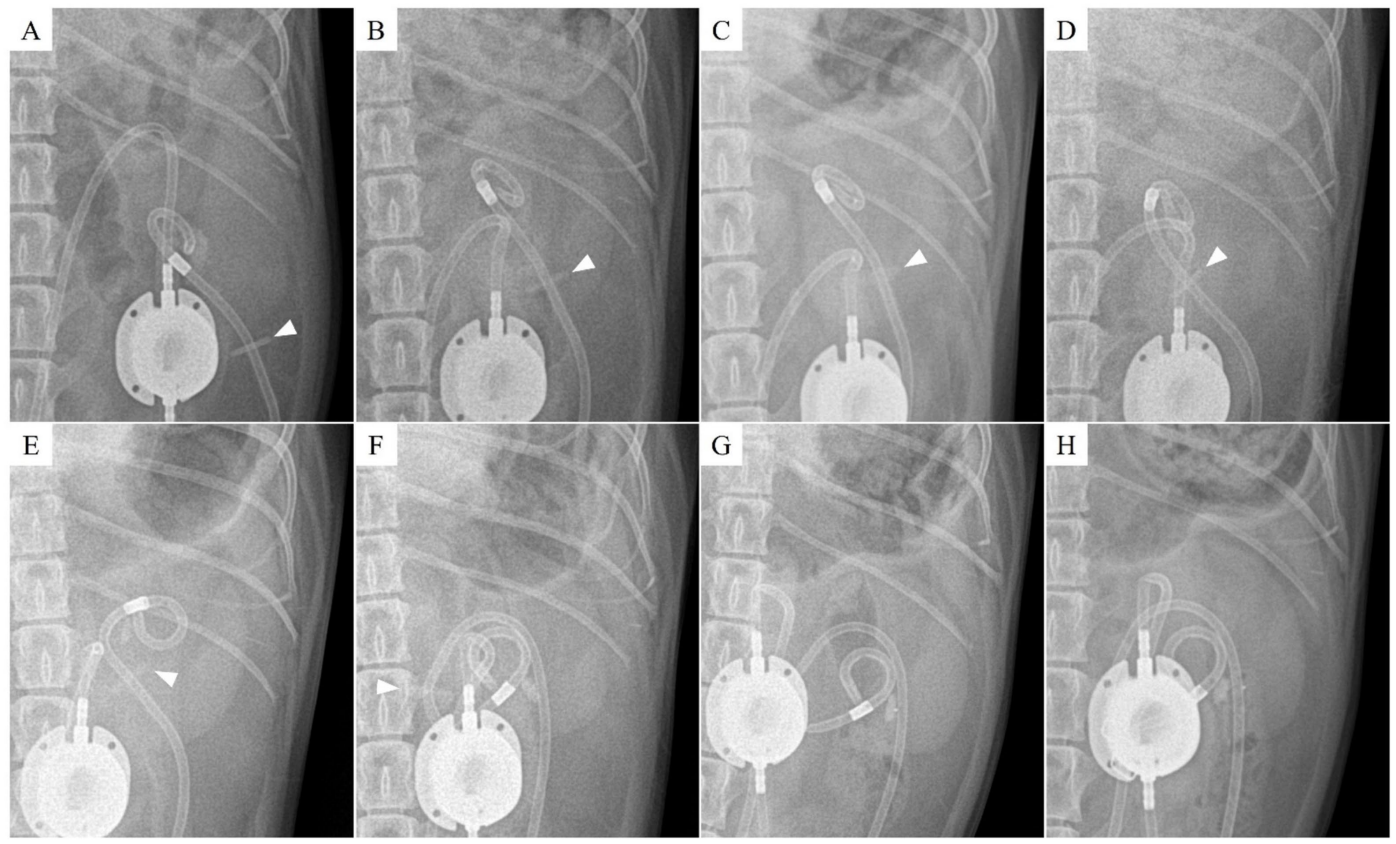
Radiographic findings of nephrostomy catheter migration. **(A)** Postoperative day (POD) 147 (before migration), **(B)** POD 321, **(C)** POD 343, **(D)** POD 408, **(E)** POD 503, **(F)** POD 857, **(G)** POD 901, and **(H)** POD 930. The Dacron cuff has migrated through the renal parenchyma into the ureter, while the pigtail end of the nephrostomy catheter has shifted into the proximal ureter. The arrowhead indicates the location of the Dacron cuff.

**Figure 3:**
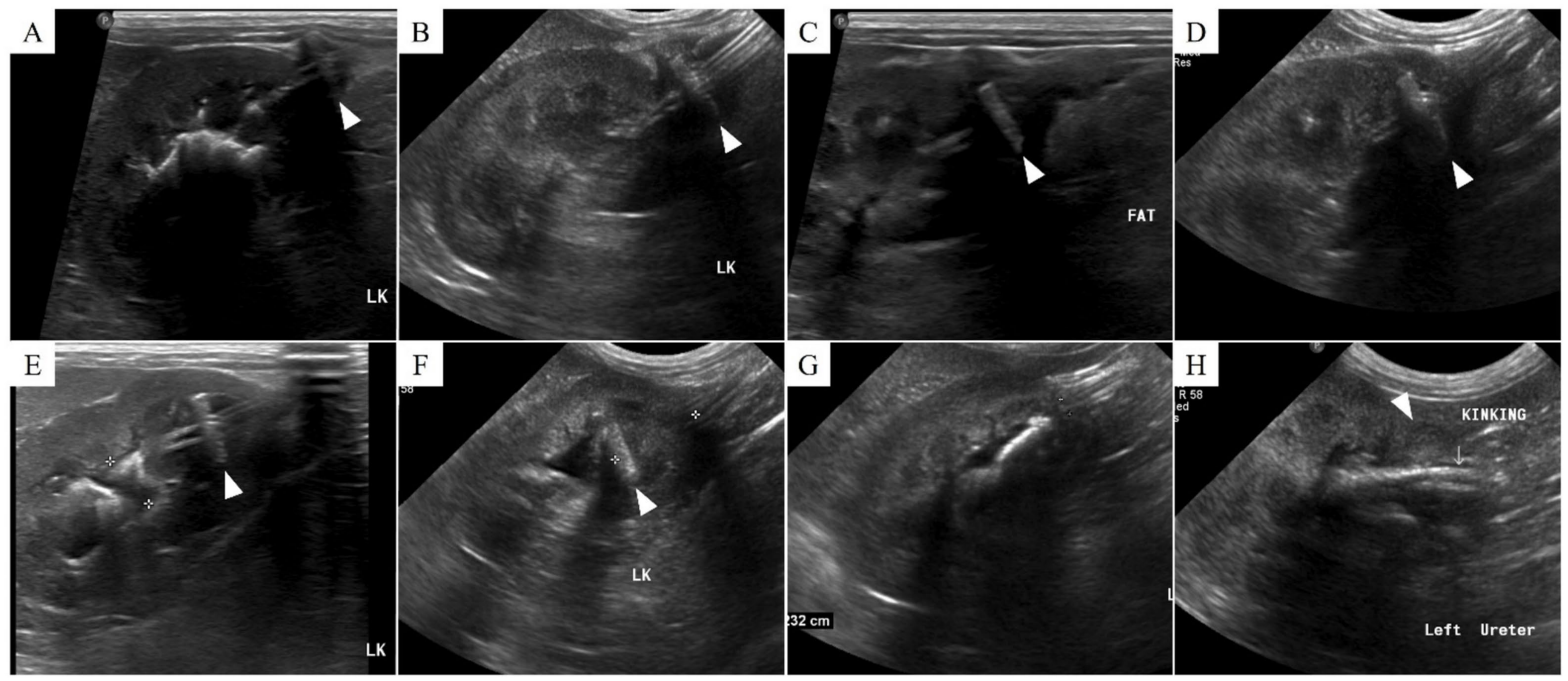
Ultrasonographic findings of nephrostomy catheter migration. **(A)** Postoperative day (POD) 148 (before migration), **(B)** POD 180, **(C)** POD 212, **(D)** POD 216, **(E)** POD 369, **(F)** POD 460, **(G)** POD 857, and **(H)** POD 901. On POD 212, low echogenic inflammatory lesions and fat edema are observed around the Dacron cuff. Subsequently, from **(C)** to **(H)**, the Dacron cuff gradually penetrates the renal parenchyma and migrates into the ureter, resulting in kinking. The arrowhead indicates the location of the Dacron cuff.

**Figure 4:**
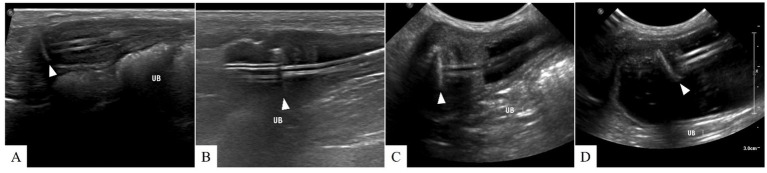
Ultrasonographic findings of cystostomy catheter migration. **(A)** Postoperative day (POD) 148 (before migration), **(B)** POD 369, **(C)** POD 654, and **(D)** POD 796. On POD 369, low echogenic inflammatory lesions and fat edema are observed around the Dacron cuff. From **(B)** to **(D)**, the cystostomy catheter migrates into the bladder lumen, resulting in severe bladder wall inflammation. No signs of urine leakage are suspected after migration. The arrowhead indicates the location of the Dacron cuff.

Inflammation was initially observed around the Dacron cuff of the nephrostomy catheter on POD 212 (Figures 2, 3). By POD 369, SUB migration into the renal parenchyma was confirmed. Additionally, on POD 369, inflammation was noted around the Dacron cuff of the cystostomy catheter (Figure 4). The bladder apex thickened, and the Dacron cuff migrated into the thickened bladder wall, eventually migrating entirely into the bladder by POD 796. Urine leakage was not suspected. By POD 901, the cuff of the nephrostomy catheter had gradually migrated through the renal parenchyma and into the ureter. On POD 901, SUB obstruction was also confirmed. No abnormalities were observed during the migration process.

On POD 740, the SUB shunting port protruded, and primary wound closure was attempted. Primary and secondary closure attempts were unsuccessful when the SUB shunting port was re-exposed on POD 896.

On POD 930, due to migration, SUB obstruction, and extrusion of the SUB shunting port, it was decided to remove the SUB. The kidney panel was within the normal range. The patient was anesthetized using the same procedure as before. The midline laparotomy uncovered omental adhesions at the cystostomy catheter insertion site, significantly thickening the bladder wall. Preoperative intravenous pyelography (IVP) verified ureteral patency, while contrast fluoroscopy via the SUB shunting port revealed no nephrostomy catheter opacification during operation, indicating that the ureter was patent. The SUB was firmly affixed to tissue, and the nephrostomy catheter had penetrated the kidney into the ureter, prompting concerns about renal injury upon removal. During the surgery, only the SUB shunting port and cystostomy catheter were excised, while the nephrostomy catheter was ligated to prevent urine leakage.

On POD 937, the kidney panel at discharge remained within the normal range (creatinine 0.81 mg/dL, BUN 14.12 mg/dL). The ultrasound revealed two ureteroliths upon SUB removal, but 133 days later (POD 1063 from the initial surgery), they had disappeared. On POD 1072, the Dacron cuff had migrated into the renal pelvis again.

## 3 Discussion

Ureteral obstruction is managed medically or surgically, with its severity and duration impacting the glomerular filtration rate (GFR) (2, 20). Traditional surgical options for ureteral obstruction include ureterostomy, ureteral reimplantation, ureteronephrectomy, and ureteral resection with anastomosis (3–7). However, these methods have high complications (30–38%) and mortality rates (18–20%) (4, 8, 9). SUB is recommended for its lower mortality rate (<5%), lower complication rate (1.4–27%), and longer median survival time (762–923 days) (8, 10–12).

Aggressive surgical intervention was deemed necessary due to imaging findings indicative of hydronephrosis, renal damage, and retroperitoneal inflammation, corroborated by serum chemistry confirming azotemia despite the absence of anuria. Given the presence of a single kidney, preserving the glomerular filtration rate was imperative. Moreover, the accompanying infection raised concerns about the progression to pyelonephritis, septicemia, and mortality (21). Retropulsion of proximal ureteroliths to the renal pelvis for pyelolithotomy was also considered (22). However, the renal pelvis presented insufficient dilation (8 mm), thereby complicating the surgical procedure (23). Additionally, stricture concerns prompted the SUB placement.

SUB complications include intermittent dysuria (12.5–38.5%), obstruction (5.3–33.3%), UTI (8–30.8%), mineralization (25%), kinking (4–12.5%), leakage (3.5%), transmural migration into the small intestine (1%) (18, 19), and intestinal perforation of the nephrostomy catheter and Dacron cuff without catheter migration (2 cats), extrusion of the SUB shunting port (1 cat) (17), and enterovesicular fistula at the cystostomy catheter site (1 cat) (12–15, 24). However, there are no reports concerning catheter migration through the renal parenchyma and bladder wall into the ureteral and bladder lumina.

SUB migration may result from a foreign body reaction, potentially involving the Dacron cuff and cyanoacrylate glue (18, 19, 25). This is similar to reports of biomaterials causing transmural migration into the gastrointestinal tract, bladder, lungs, and trachea due to foreign body reaction (26–28). Late Dacron patch inflammatory complications in humans have been reported up to 7 years post-implantation, suggesting secondary foreign body reactions from the Dacron cuff in the abdominal cavity could cause transmural migration (25). In this patient, inflammation likely first developed around the Dacron cuff, possibly triggering a foreign body reaction that contributed to catheter migration.

Prior to SUB placement surgery, this patient had already been diagnosed with pyelonephritis caused by both rods and cocci. Postoperatively, bacterial biofilm formation on the surface of the SUB device may have contributed to persistent infection, making it difficult to control. Consequently, chronic UTI may have potentially facilitated the SUB migration (17–19, 26, 27).

In this patient, despite two ureteroliths remaining, IVP confirmed ureteral patency, facilitating SUB removal. When infected, biofilm on the SUB may require removal of the SUB and high-dose antibiotic therapy (29–31). Even after SUB replacement, persistent infections in surrounding tissues might result in secondary infection (32). Furthermore, its removal or replacement should be decided based on ureteral patency (18). Focal inflammation and ureteral muscle spasm can exacerbate ureteral obstruction (33), and half of SUB reobstruction cases regained ureteral patency (13, 20).

There were no signs of suspected urine leakage, likely due to severe omental adhesions and gradual migration. However, in cases of SUB migration, it is crucial to confirm the absence of any leakage, whether from the intestine, bladder, or other sources, as cases of intestinal leakage with septic peritonitis have been reported (25).

A limitation of this case is that the nephrostomy catheter was not removed during the second surgery. Despite our recommendation for revision surgery to remove the remaining nephrostomy catheter, the procedure was not performed. The owner declined it, as the patient was clinically stable. However, in cases of migration, it is generally preferable to remove the entire SUB from the outset.

In conclusion, the long-term management of SUB devices should include monitoring for potential complications, such as the gradual migration of the nephrostomy and cystostomy catheters into the renal pelvis and bladder lumen, as observed in this patient. Prompt identification of this complication is crucial for reducing morbidity and facilitating timely, appropriate intervention.

## Data Availability

The original contributions presented in the study are included in the article/Supplementary material, further inquiries can be directed to the corresponding author.
