# Supplementary material for: Case Report: Long-term complications of subcutaneous ureteral bypass migration in an adult female Papillon
Source: Front Vet Sci. 2025 Mar 12;12:1543299. doi: 10.3389/fvets.2025.1543299 (PMC11938841; doi:10.3389/fvets.2025.1543299)
Supplement: Supplementary file 1 [file Data_Sheet_1.docx]

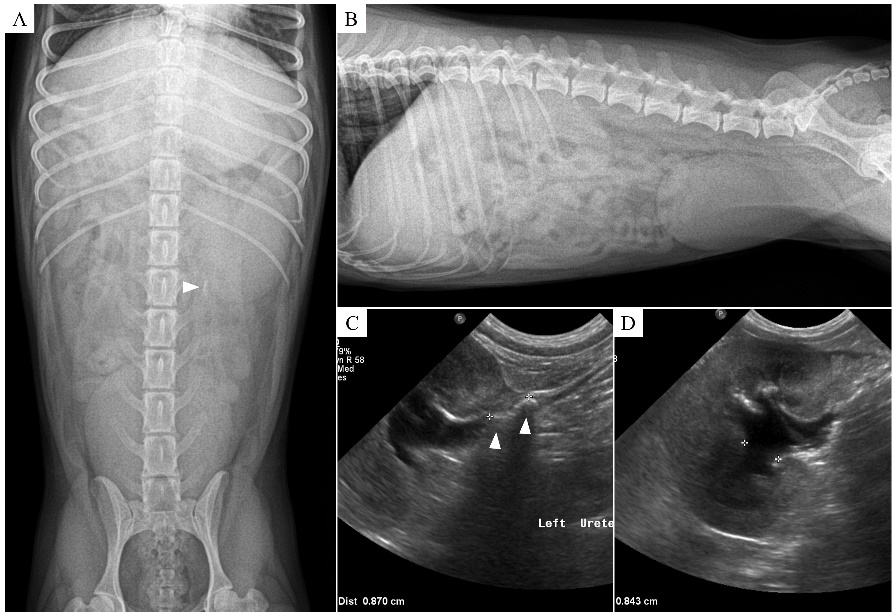


Supplementary file 1. Radiological and ultrasound findings at the time of presentation. (A, B) Radiographs demonstrate an enlargement of the left kidney and the presence of ureteroliths (arrowhead). (C) Ultrasound reveals two ureteroliths measuring 6–8 mm in the left proximal ureter. (D) Approximately 8 mm of renal pelvis dilation due to ureteroliths, increased parenchymal echogenicity, and surrounding fat thickening and echogenicity are observed, suggesting the presence of hydronephrosis, renal damage, and retroperitoneal inflammation. The arrowhead indicates the location of the ureteroliths.


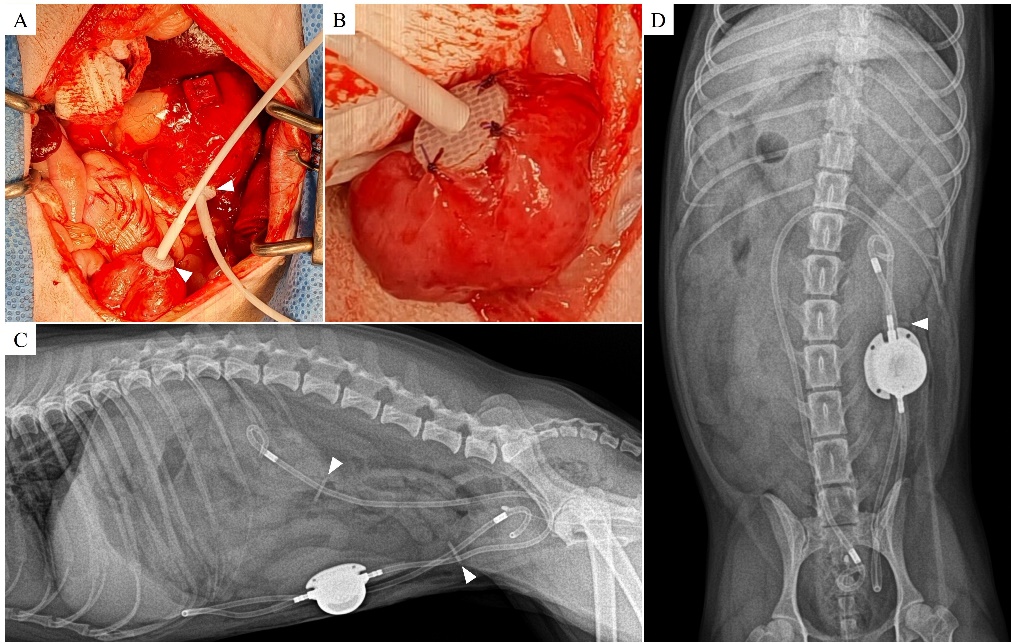


Supplementary file 2. Surgical and radiological findings during the subcutaneous ureteral bypass placement. (A) The nephrostomy and cystostomy catheters are secured to the caudal pole of the kidney and apex of the urinary bladder, respectively, using a Dacron cuff (arrowhead) and cyanoacrylate glue for subcutaneous ureteral bypass (SUB) placement. (B) The Dacron cuff is affixed using cyanoacrylate glue and sutured with simple interrupted sutures. (C, D) Postoperative radiographs confirm the correct placement of the SUB.
